# Multiple Sclerosis: Enzymatic Cross Site-Specific Hydrolysis of H1 Histone by IgGs against H1, H2A, H2B, H3, H4 Histones, and Myelin Basic Protein

**DOI:** 10.3390/biom11081140

**Published:** 2021-08-02

**Authors:** Georgy A. Nevinsky, Svetlana V. Baranova, Valentina N. Buneva, Pavel S. Dmitrenok

**Affiliations:** 1 Siberian Division, Institute of Chemical Biology and Fundamental Medicine, Russian Academy of Sciences, Lavrentiev Ave. 8, 630090 Novosibirsk, Russia; swb@ngs.ru (S.V.B.); buneva@niboch.nsc.ru (V.N.B.); 2 Far East Division, Pacific Institute of Bioorganic Chemistry, Russian Academy of Sciences, 690022 Vladivostok, Russia; paveldmt@piboc.dvo.ru

**Keywords:** human blood sera antibodies, multiple sclerosis patients, catalytic antibodies-abzymes, hydrolysis of H1 histone, IgGs against H1, H2A, H2B, H3, H4 histones, myelin basic protein

## Abstract

Histones play a key role in chromatin remodeling and gene transcription. Further, free histones in the blood act as damage-associated molecules. Administration of histones to animals results in systemic inflammatory and toxic effects. Myelin basic protein is the principal constituent element of the myelin-proteolipid sheath of axons. Abzymes (antibodies with catalytic activities) are the original features of some autoimmune diseases. In this study, electrophoretically homogeneous IgGs against H1, H2A, H2B, H3, and H4 histones and myelin basic protein (MBP) were isolated from the blood sera of multiple sclerosis (MS) patients by several affinity chromatographies. Using MALDI mass spectrometry, the sites of H1 histone cleavage by IgGs against H1, H2A, H2B, H3, H4, and MBP were determined. It was shown that IgGs against H1 split H1 at 12 sites, while the number of cleavage sites by abzymes against other histones was lower: H2A (9), H2B (7), H3 (3), and H4 (3). The minimum rate of H1 hydrolysis was observed for antibodies against H3 and H4. A high rate of hydrolysis and the maximum number of H1 hydrolysis sites (17) were found for antibodies against MBP. Only a few sites of H1 hydrolysis by anti-H1 antibodies coincided with those for IgGs against H2A, H2B, H3, H4, and MBP. Thus, the polyreactivity of complexation and the enzymatic cross-activity of antibodies against H1, four other histones, and MBP have first been shown. Since histones act as damage molecules, abzymes against histones and MBP can play a negative role in the pathogenesis of MS and probably other different diseases as well.

## 1. Introduction

Antibodies (Abs) against stable analogs of reaction transition states and natural auto-antibodies with enzymatic activities are known as “abzymes,” and they are decently described in the literature [1,2,3,4,5,6]. The spontaneous and antigen-accelerated development of autoimmune diseases (AIDs) leads to the production of auto-antibodies-abzymes (ABZs) against lipids, polysaccharides, peptides, proteins, DNAs and RNAs, and their complexes. In the blood sera of autoimmune disease patients, many different ABZs directly against various antigens mimicking transition states of chemical reactions have been found. Secondary anti-idiotypic auto-ABZs to active sites of canonical enzymes have also been found, whose forms may be explained by Erne’s model of the anti-idiotypic network [7]. The appearance of ABZs in the blood is a very clear sign of the occurrence of autoimmune processes in humans and mammals [1,2,3,4,5,6]. To date, IgGs or IgMs, and IgAs splitting DNAs, RNAs [8,9,10,11,12], oligosaccharides [13,14,15], oligopeptides, and proteins [16,17,18,19,20,21,22,23] have been found in the blood of patients with autoimmune and various viral diseases [1,2,3,4,5,6].

Some healthy humans and mammals produce ABZs with low vasoactive intestinal peptide- [16], thyroglobulin- [18], and polysaccharide-hydrolyzing [13,14,15] activities, but usually, healthy humans and patients with some diseases characterizing insignificant autoimmune reactions lack abzymes [1,2,3,4,5,6]. Nonetheless, germline antibodies of healthy humans could demonstrate amyloid-directed and superantigen-directed enzymatic activities [24,25].

MBP is the main component of the myelin-proteolipid sheath of axons. The protein-specific abzymes against MBP can attack and hydrolyze the MBP of the myelin sheath of axons, playing a very negatory role in MS pathogenesis due to their disrupting of nerve impulse conduction. [1,2,3,4,5,6,21,22]. Histones and their different post-translational forms bear a vital role in the functioning and remodeling of chromatin. Free extracellular histones act as damage molecules, causing systemic toxic effects through inflammatory pathways and the activation of Toll-like receptors [26]. Abzymes that hydrolyze MBP and five histones have been detected in the blood of HIV-infected [21,22,27,28,29,30,31,32,33,34], SLE [35], and MS [36] patients, and in mice with experimental encephalomyelitis [37]. In autoimmune diseases patients, many anti-DNA and anti-histones Abs are directed against histone-DNA complexes appearing in the blood due to internucleosomal cleavage during apoptosis [38]. The existence of enzymatic cross-reactivity of ABZs against MBP and histones is dangerous to humans because histones, caused by cell apoptosis, occur constantly in human blood. Considering this, the analysis of the possible catalytic cross-reactivity of antibodies against MBP and histones is very important.

The unspecific complexation of some antigens with antibodies against foreign ligands is a widely distributed phenomenon [39,40,41,42]. Specific for various substrates, classic enzymes usually catalyze only one chemical reaction [43,44,45]. All described to date ABZs against different proteins could usually split only their specific proteins [1,2,3,4,5,6]. The first examples of catalytic cross-reactivity were anti-MBP IgGs and antibodies against H1, H2A, H2B, H3, and H4 histones from the sera of HIV-infected patients [32,33,34]. At the same time, an analysis of the possible polyreactivity of complexation and catalytic cross-reactivity of antibodies against the five histones themselves has not yet been carried out. We have suggested that if antibodies against H1, H2A, H2B, H3, and H4 histones have enzymatic cross-reactivity with antibodies against MBP, then they potentially can hydrolyze not only their specific histone but other histones as well.

In this work, an analysis of the ability of the antibodies of MS patients against H1, H2A, H2B, H3, and H4 histones and MBP to hydrolyze H1 histone was performed for the first time. It was shown that not only abzymes against these proteins of HIV-infected patients [32,33,34], but also those of patients with multiple sclerosis, have catalytic cross-reactivity. Moreover, it was also shown that abzymes against all five histones are capable of hydrolyzing histone H1 with different efficiency and in different sites.

## 2. Material and Methods

### 2.1. Chemicals, Donors, and Patients

All chemicals used, including five homogeneous human histones and an equimolar mixture of five histones (H1, H2A, H2B, H3, and H4), were from Sigma (St. Louis, MO, USA). Superdex 200 HR 10/30 and Protein G-Sepharose columns were obtained from GE Healthcare (GE Healthcare, New York, NY, USA). Human MBP was perched from the Molecular Diagnostics and Therapy Center of DBRC (Moscow, Russia). Histone- and MBP-Sepharose columns were prepared according to the manufacturer’s protocol using BrCN-activated Sepharose (Sigma), MBP, five individual histones, or their mixtures.

Evidence that multiple sclerosis patients hydrolyze five histones and MBP has been previously published using IgGs from the blood of 59 patients [21,22,31]. Patient data are given in Supplementary Table S1. The diagnosis of multiple sclerosis was established according to the classification of McDonald [46] by specialists from the multiple sclerosis center (Novosibirsk Medical University). Disease severity of all 59 MS patients was scored using Kurtzke’s Expanded Disability Status Scale (EDSS) [47]. The patients at entry had no symptoms of any infections. None of the MS patients at the time of sample collection had received any anti-disease therapies during the 6 months before the study.

The protocol of blood sampling was confirmed by the local human ethics committee (Novosibirsk State Medical University, Novosibirsk, Russia; number 105-HIV; 07. 2010). This ethics committee supported this study based on the guidelines of the Helsinki ethics committee. All patients made a written agreement to donate blood for scientific purposes.

In this work, we used an equimolar mixture of 15 of the 59 IgGs described earlier [31] with high activity in the hydrolysis of five histones and MBP.

### 2.2. Antibody Purification

Electrophoretically homogeneous preparations of IgGs were obtained from the blood of MS patients by sequential affinity chromatography of the blood serum proteins, first on Protein G-Sepharose and additionally by gel filtration on a Superdex 200 HR 10/30 column, as in [31,32,33,34]. To protect IgG preparations from bacterial and viral contamination, they were filtered through a Millex filter (pore size 0.1 µM). After 6–7 days of storage at 4 °C for refolding, the IgGs were used in different assays. SDS-PAGE analysis of Abs for homogeneity was carried out in 4–17% gradient gels (0.1% SDS), and proteins were visualized by silver staining, as in [31,32,33,34].

Earlier, using eluates of 3–4-mm cross-sections of the gel longitudinal slices after SDS-PAGE of antibodies, it has been shown that IgGs of MS patients do not contain any canonical protease impurities [31]. Protease activity was found only in the eluates of the gel fragments corresponding to IgGs.

### 2.3. Affinity Chromatography of IgGs on MBP- and Histones-Sepharose

To isolate antibodies against various histones, we used a mixture of 15 Abs (IgG_mix_) that exhibit high activity in the hydrolysis of histones and MBP [31]. Removal of all Abs against five histones from homogeneous polyclonal IgG_mix_ having no impurities of any canonical proteases was carried out using MBP-Sepharose bearing immobilized MBP, equilibrated in buffer A (20 mM Tris-HCl, pH 7.5). After IgGs loading, to obtain the fraction containing IgGs against MBP, the column was washed to zero optical density (A_280_) with buffer A. Adsorbed IgGs against MBP were eluted using buffer A containing NaCl (0.2 M), and finally were specifically eluted by 3.0 M NaCl and 0.1 M glycine-HCl, pH 2.6. The fractions eluted from MBP-Sepharose with 3.0 M and acid buffer were used for additional purification from potentially possible impurities of IgGs against 5 histones. They were applied on the column of histone5-Sepharose (immobilized mixture of 5 histones). The fraction of Abs eluted upon the loading was named and used as anti-MBP IgGs.

The fraction eluted from MBP-Sepharose at loading (containing a mixture of IgGs against 5 histones) was used for purification of the IgGs against five individual histones using coherently Sepharose-containing immobilized H1 (H1-Sepharose), H2A (H2A-Sepharose), H3 (H3-Sepharose), and H4 (H4-Sepharose) histones. Antibodies with no affinity for the previous sorbent were applied to the next sorbent. All chromatographies were performed as in the case of MBP- and histone5-Sepharose. IgGs against H1-H4 histones were specifically eluted with buffer (pH 2.6). These fractions were named anti-H1, anti-H2A, anti-H2B, anti-H4, and anti-H4 IgGs, respectively.

### 2.4. Proteolytic Activity Assay

The reaction mixtures (10–15 µL) contained 20 mM Tris-HCl (pH 7.5), 0.7 mg/mL H1 histone or 1.0 mg/mL MBP, and 0.01–0.1 mg/mL IgGs against one of five histones, H1–H4, or MBP. The mixtures were incubated for 1–24 h at 37 °C. The efficiency of histone H1 and MBP splitting was analyzed by standard SDS-PAGE using 15% gels under nonreducing conditions (in the absence of DTT), as in [27,28,29,30,31,32,33,34,35]. The products of H1 and MBP hydrolysis were revealed using Coomassie Blue. The gels after painting were scanned and quantified using Image Quant v5.2 software, as in [31]. The efficiency of protein hydrolysis was assessed by their decrease in comparison with the control-incubation of the proteins in the absence of antibodies.

### 2.5. MALDI-TOF Analysis of Abs-Dependent H1 Histone Hydrolysis

H1 hydrolysis by IgGs against five histones (H1–H4) was performed using MALDI analysis. The analysis was carried out using the Reflex III system from Bruker Company (Frankfurt, Germany): 337-nm nitrogen laser VSL-337 ND, 3 ns pulse duration. Mixtures (10 µL) containing 20 mM Tris-HCl (pH 7.5), 0.7 mg/mL one of histones and 0.04 mg/mL one of IgG preparation were incubated during 0–24 h. To 1.2 µL of the sinapinic acid matrix mixed with 1.2 µL of 0.2% trifluoroacetic acid, 1.2 µL of the solutions containing H1 before or after incubation with various IgGs were added; 1–1.2 µL of these mixtures were applied on the MALDI plates, which then were air-dried. Calibrations of all MALDI spectra were performed using standard protein mixtures II and I (Germany, Bruker Daltonic) in the internal and/or external calibration modes. The analysis of peptide MMs corresponding to specific sites of H1 hydrolysis by different IgGs was performed using Protein Calculator v3.3 (Scripps Research Institute; La Jolla, CA, USA).

### 2.6. Analysis of Sequence Homology

The analysis of homology between histones and MBP sequences was carried out using *lalign* (http://www.ch.embnet.org/software/LALIGN_form.html) (accessed on Internet).

### 2.7. Statistical Analysis

The results are given as mean ± S.D. of 7–10 independent MALDI specters for each sample of the different IgGs.

## 3. Results

### 3.1. Purification of Antibodies

In this work, we used previously described electrophoretically homogeneous polyclonal IgGs from the sera of 15 MS patients isolated by sequential chromatography of the serum proteins, first on Protein G-Sepharose in conditions allowing for the removal of non-specifically bound proteins [31,32,33,34]. Then, IgGs were subjected to FPLC gel filtration under rigid conditions (pH 2.6), destroying immune complexes according to [31,32,33,34]. To analyze the “average” site-specific hydrolysis of H1 histone by IgGs against five histones (H1–H4), we prepared a mixture of equal amounts of 15 IgGs (IgG_mix_), which demonstrated high activities in the splitting of H1 histone and MBP. Any possible artifacts due to traces of contaminating canonical proteases were excluded earlier [31]; the IgG_mix_ was separated by SDS-PAGE, and its histone- and MBP-hydrolyzing activities were detected in only one protein band corresponding to IgG_mix_. Then, we isolated IgGs against MBP by IgG_mix_ affinity chromatography on MBP-Sepharose. IgGs nonspecifically bound to the sorbent were first eluted with 0.2 M NaCl. Specific IgGs against MBP were eluted with 3.0 M NaCl and acidic buffer (pH 2.6). For additional purification from potential impurities against five histones, a mixture of these IgG fractions was passed through histone5-Sepharose. The fraction released upon loading onto the column was further used as anti-MBP IgGs.

The fraction eluted from MBP-Sepharose on loading was used to isolate IgGs against five individual histones. This fraction was passed once more through MBP-Sepharose and then used to isolate antibodies against five individual histones. For this, it was applied sequentially to five sorbents with immobilized H1, H2A, H2B, H3, and H4 histones. Fractions without affinity for the previous sorbent, eluted upon loading, were used for each subsequent chromatography. Finally, we obtained five preparations of antibodies against five individual histones: anti-H1, anti-H2A, anti-H2B, anti-H3, and anti-H4 IgGs.

### 3.2. SDS-PAGE Analysis of Histones and MBP Hydrolysis

**Figure 1:**
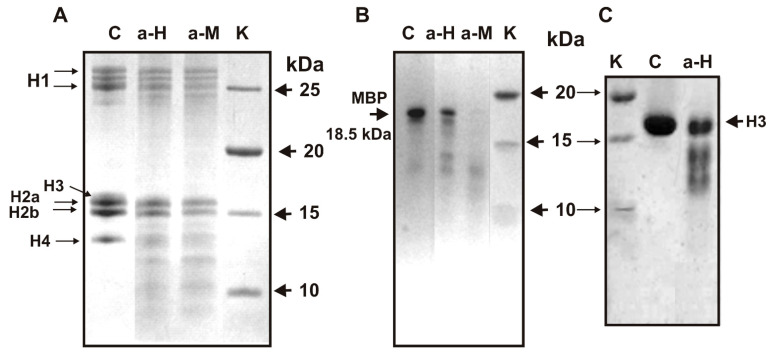
SDS-PAGE analysis of five H1-H4 histones hydrolysis by IgGs against five histones (lane a-H) and MBP (lane a-M) (**A**) as well as MBP by anti-histones (lane a-H) and anti-MBP IgGs (lane a-M) (**B**). Hydrolysis of homogeneous H3 histone by IgGs against five histones (**C**). Lane C correspond to hive histones (**A**), and MBP (**B**) and homogeneous H3 (**C**) incubated in the absence of IgGs. Lane K corresponded to proteins with known molecular masses (**A**–**C**). Five histones, MBP and homogeneous H3 in the absence and in the presence of IgGs (0.03 mg/mL) were incubated for 10 h.

As shown earlier, polyclonal antibodies from the blood of HIV-infected patients [27,28,29,30] and patients with MS effectively hydrolyze both five histones [31] and MBP [21,22]. Moreover, antibodies from the blood of HIV-infected patients against five histones and MBP possess not only the ability of polyspecific complexation but are also capable of efficiently cross-reactively hydrolyzing both five histones and MBP [31,32,33,34]. An interesting question was whether IgGs from the blood of MS patients against histones are also capable of hydrolyzing both five histones and MBP, and vice versa. To analyze this kind of catalytic cross-reactivity, we used the total fraction of anti-histone IgGs (specifically eluted from histone5-Sepharose) and anti-MBP antibodies (specifically eluted from MBP-Sepharose). As can be seen from Figure 1A, anti-histone and anti-MBP IgG hydrolyze all five histones with comparable efficiency. The MMs of H3 and H2A and their electrophoretic mobility are so close that they cannot be separated efficiently (Figure 1A). Therefore, to confirm that the hydrolysis of H3 is more reliable, we performed homogeneous H3 histone hydrolysis using the total fraction of anti-histone IgGs (Figure 1C).

Electrophoretically homogeneous preparations of human MBP, unfortunately, were not available. Due to cDNA alternative splicing and different partial MBP hydrolysis in the brains of various humans, these protein preparations could contain several related forms (18.5, 17.5, and ≤14.0 kDa) and products of their hydrolysis [21,22]. Figure 1B demonstrates MBP hydrolysis by anti-MBP and anti-histone IgG preparations. Line C of Figure 1B demonstrates the starting MBP preparation heterogeneity containing mainly 18.5 kDa protein forms. After 10 h of incubation with both IgG preparations, all MBP forms decreased remarkably compared to the control (lane C). These data may potentially indicate that IgGs against histones and MBP from the sera of MS patients could possess a known phenomenon of polyreactivity in complex formation [39,40,41,42] and mutual catalytic cross-reactivity in MBP- and histone hydrolysis. These data, however, do not provide absolute evidence of catalytic cross-reactivity between IgGs against five histones and MBP because, in spite of their purifications by several affinity chromatographies, it cannot be ruled out that the IgGs obtained nevertheless potentially could contain small impurities of alternative Abs. The best evidence of cross-catalytic activities may be obtained from an obvious difference in the specific sites of the hydrolysis of histones by IgGs against these five histones and against MBP. However, the main objective of this study was not only to analyze the possibility of cross-catalyzing the hydrolysis of histones and MBP by antibodies against these proteins, but for the first time to investigate, using the example of antibodies from MS patients, whether there is cross-catalytic activity of antibodies against five histones. In this work, we analyzed the possibility of the hydrolysis of histone H1 with specific Abs against five histones (H1–H4) and MBP.

### 3.3. MALDI Analysis of H1 Histone Hydrolysis

**Figure 2:**
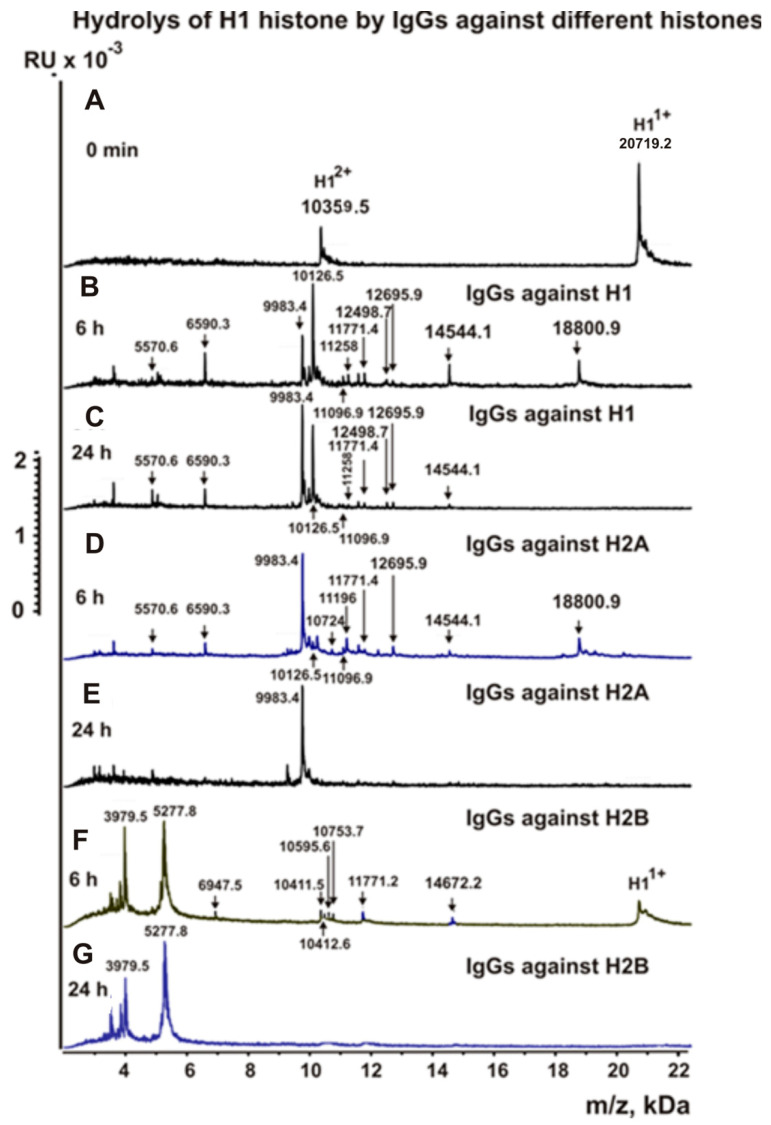
MALDI spectra (**A**–**G**) corresponding to hydrolysis of H1 histone (0.7 mg/mL) over time (0–24 h) in the presence of IgGs (0.04 mg/mL) against H1 (**B**,**C**), H2A (**D**,**E**), and H2B (**F**,**G**).

The IgG fractions with high affinity to five individual histones and to MBP were used to reveal the cleavage sites of histone H1 by MALDI TOFF mass spectrometry. After the addition of these IgGs (Figure 2A), H1 histone was nearly homogeneous: there were only two signals of its one- (*m*/*z* = 20719.2 Da) and two-charged ions (*m*/*z* = 10359.6 Da).

H1 hydrolysis assays were performed with IgGs against five histones and MBP after 3, 6, and 24 h of incubation. Almost all the main peaks corresponding to different sites of H1 hydrolysis by IgGs against H1 histone are clearly visible after 6 h of hydrolysis (Figure 2B). Incubation of mixtures for 24 h led to complete hydrolysis of initial H1 and the formation of its different fragments (Figure 2C). Based on the analysis of peaks in 10 spectra, 13 sites of the hydrolysis were identified, three of which are major (L94-A95, K131-A132, and K135-K136), six moderate (A88-S89, R93-L94, K96-S97, P100-K101, S103-V104, and T109-K110), and four minor splitting sites (V75-T76, A117-T118, A128-A129, and P141-V142).

The H1 hydrolysis assay with anti-histone H2A antibodies was carried out in a similar manner to the splitting of H1 with anti-H1 IgGs. Figure 2D,E show MALDI spectra of the products of the reaction mixture after hydrolysis of H1 histone with anti-H2A IgGs for 6 and 24 h. These antibodies effectively cleave H1 to very small peptides in 24 h (<5 kDa) with the formation of only one more stable product observed (9983.4 Da; Figure 2E). Based on the analysis of 10 spectra, 9 hydrolysis sites were reliably detected: one major (V104-A105), five moderate (R93-L94, L94-A95, A117-T118, K127-A128, and A132-P133), and three minor (R46- Q47, S103-V104, T109-K110) sites.

Anti-H2B histone antibodies hydrolyzed H1 with approximately the same efficiency as anti-H1 and anti-H2A antibodies, and after 24 h of incubation, no peak of initial histone H1 was found (Figure 2F,G). Interestingly, in this case of H1 hydrolysis by anti-H2B antibodies, other sites in comparison with those for anti-H1 and anti-H2A antibodies were mainly found. Seven reliably detectable hydrolysis sites were found, among which one was major (A156-K157) and six were minor sites (Q82-T83, R93-L94, S97-D98, P100-K101, K127-A128, K136-P137) (Figure 2F).

**Figure 3:**
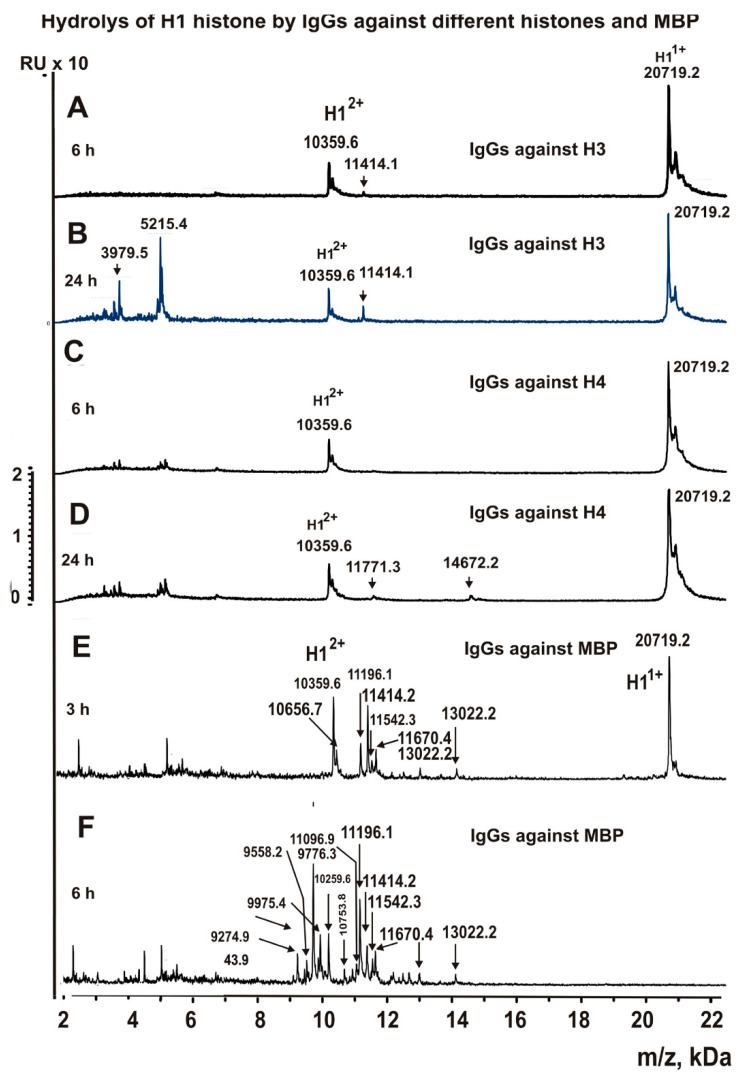
MALDI spectra (**A**–**G**) corresponding to H1 histone (0.7 mg/mL) hydrolysis over time (6–24 h), in the presence of IgGs (0.04 mg/mL) against H3 (**A**,**B**), H4 (**C**,**D**), and MBP (**E**,**F**).

Antibodies against histone H3 also cleaved H1, but at a rate approximately 7–10-fold lower than that of the anti-H1, anti-H2A, and anti-H2B abzymes (Figure 3A,B). After 6 h of incubation, only one detectable product of the hydrolysis was detected (Figure 3A). After 24 incubation, two additional reliably tested products of histone splitting appeared.

Based on the totality of 10 spectra, only three H1 cleavage sites by anti-histone H3 antibodies were found. One of the sites should be considered as major (A145-K146), one moderate (A156-K157), and one minor (F106-K107).

The abzymes against histone H4 hydrolyzed H1 very slowly. Only after 24 h of incubation were several significant peaks of H1 hydrolysis by these antibodies found (Figure 3C,D). Ultimately, three reliably tested sites for hydrolysis of H1 by anti-H4 antibodies were identified. Two sites were classified mostly as minor sites (K127-A128, and K136-P137) and only T109-K110, the peak corresponding to which appears first, can be classified as a moderate hydrolysis site.

It should be noted that anti-MBP antibodies efficiently hydrolyze all five histones (Figure 1A). Figure 3B,D show the spectra of the products of H1 hydrolysis by antibodies against MBP for 3 and 6 h of hydrolysis. It can be seen that, especially after 6 h, a large number of histone H1 hydrolysis products were formed. The anti-MBP abzymes hydrolyzed H1 very efficiently. After 6 h of incubation, 17 reliable sites of H1 hydrolysis were found. Four of them were major (16K-17A, V104-A105, F106-K107, and E112-I113), two were moderate (14R-15A, K107-K108) and 11 were minor, but the reliably tested sites were the following: P12-K13, K19- K20, K20-S21, S21-T22, P100-K101, K108-T109, K115-V116, V116-A117, A117-T118, K120-K121, and K131-A132.

**Figure 4:**
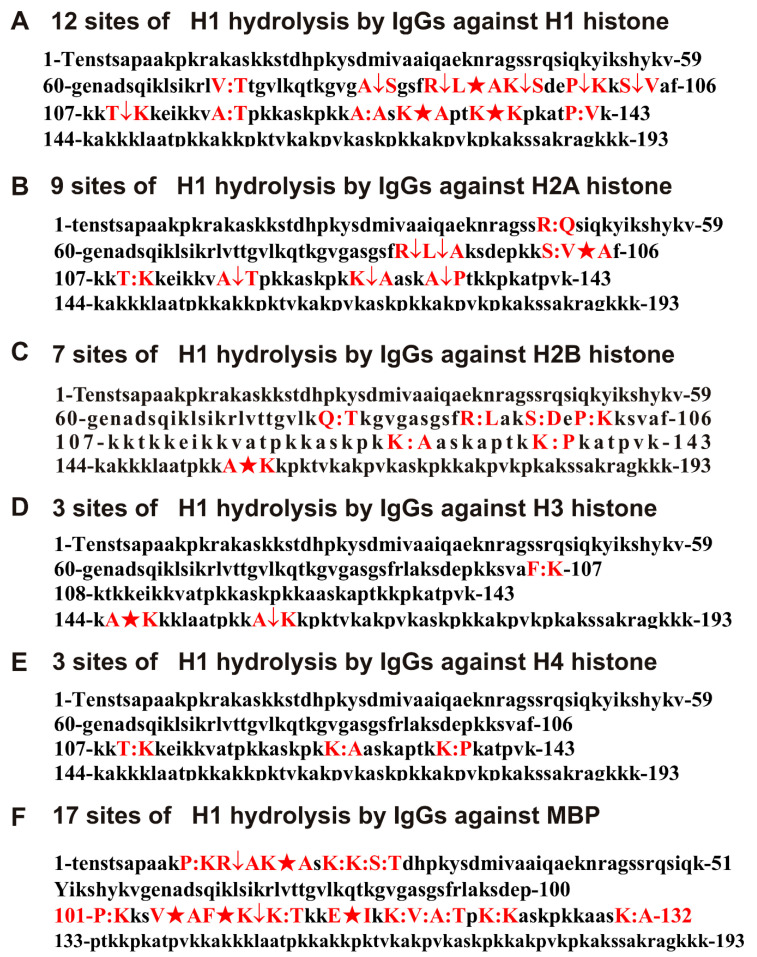
Sites of H1 hydrolysis by IgGs against H1 (**A**), H2A (**B**), H2B (**C**), H3 (**D**), H4 (**E**), and MBP (**F**). Major sites of H1 splitting are shown by stars (★), moderate ones by arrows (↓), and minor sites of the cleavages by colons (:) (**A**–**F**).

Figure 4 summarizes the data on the sites of H1 hydrolysis by antibodies against all 5 histones and MBP. Major hydrolysis sites are indicated by stars (★), moderate by an arrow (↓), and minor by colons (:).

As can be seen from Figure 4, antibodies against five histones and MBP strongly differ in the number, cleavage efficiency, and position of hydrolysis sites in the histone H1 protein sequence. To simplify the analysis of the overlapping and different hydrolysis sites of the six antibody preparations, all data are collected in Table 1.

As can be seen from Figure 4F and Table 1, one of the H1 hydrolysis sites clusters in the case of anti-MBP antibodies, which contains 6 hydrolysis sites, located from P12 to T22 amino acid residues (AA). In this zone of the histone H1 protein sequence, there are no hydrolysis sites in the case of abzymes against five histones (Figure 4A–E, Table 1). It should be noted that the sites of histone H1 hydrolysis by antibodies against this histone are located mainly in two clusters: the first, V75–V104 (7 hydrolysis sites), and the second, A117-B142 (5 hydrolysis sites).

In the zone of the first cluster, there are four cleavage sites of H1 by antibodies against H2A (Table 1). At the same time, there is a coincidence of only two hydrolysis sites (R93-L94 and S103-V104) for antibodies against H1 and H2A abzymes. In the same region of the H1 sequence, there are only four sites of its hydrolysis by anti-H2B abzymes, and only one site (R93-L94) is common with that of antibodies against H1 and H2A abzymes. Interestingly, antibodies against H3 and H4 hydrolyze H1 histone very weakly. At the same time, in the zone of the first cluster from P12 to T22, the sites of H1 hydrolysis by these antibodies are completely absent. For antibodies against histones H3 and H4, only three H1 hydrolysis sites were reliably detected. Moreover, these sites are different, and they do not coincide with the sites of hydrolysis by antibodies against four other histones and against MBP, except for the site K127-A128, which is common for antibodies against H4, H2A, and H2B (Table 1).

In general, the sites of H1 hydrolysis by antibodies against five histones differ significantly in their position in the protein sequence and in the efficiency of the hydrolysis. It should be noted that the major H1 hydrolysis sites for IgGs against various histones are also basically different: anti-H1 (R94-A95, K131-A132, K135-K136); anti-H2A (V104-A105); anti-H2B (A156-K157), anti-H3 (A145-K146). For anti-H4, a well-pronounced major site of hydrolysis was not revealed; for the first 3 h, and only after 6 h of hydrolysis, a peak corresponding to the site T109-K110 appears, which may be marked as the average site.

## 4. Discussion

As noted above, the complexation polyreactivity of antibodies is a widespread phenomenon [39,40,41,42]. Even to a certain extent, related compounds can form complexes with the same antibodies. This leads to the fact that during affinity, chromatography on a sorbent, not only antibodies against this antigen but also antibodies to compounds with structural elements of this antigen, can bind to the immobilized antigens [39,40,41,42]. However, the affinity of Abs for foreign molecules is usually significantly lower than for their own antigens. This leads to the fact that antibodies against foreign antigens are usually eluted during affinity chromatography when using NaCl at a concentration of up to 0.1–0.15 M [1,2,3,4,5,6,32,33,34]. Considering this, when isolating IgGs against five individual histones and MBP, we eluted antibodies nonspecifically bound to the immobilized six antigens using 0.2 M NaCl. During further additional purification of the antibodies against histones and against MBP, they were additionally passed through alternative affinity sorbents. Eventually, antibody fractions were obtained against five individual histones and MBP.

As shown earlier in [31], the MS patients’ antibody preparations used by us do not contain any admixtures of any canonical proteases. In addition, the same conclusion can be drawn based on the analysis of histone H1 hydrolysis sites with Abs against five histones and MBP. As is known, trypsin cleaves proteins after the residue of lysine (K) and arginine (R). The H1 sequence contains 67 sites for potential hydrolysis of this histone by trypsin. However, the number of sites of H1 hydrolysis by all used preparations of IgGs after K and R varies mainly from 0 (anti-H3 IgGs) to 2–3; only in the case of Abs against H1 and MBP, there are more: 4 and 7, respectively. Chymotrypsin breaks down proteins after aromatic amino acids (F, Y, W). There are five potential such sites for hydrolysis of H1 histone by chymotrypsin. One site of hydrolysis after F was found in the case of anti-H3 (F106-K107; minor site) and anti-MBP (F106–107K; major site) IgGs (Table 1). In this regard, the data of [48] should be noted: when using a large number of monoclonal antibodies of patients with SLE, this study showed that their active centers could correspond to serine, thiol, or metal-dependent proteases. However, in contrast to canonical proteases, antibodies hydrolyze proteins mainly in their clusters that correspond to antigenic determinants [1,2,3,4,5,6,27,28,29,30,31,32,33,34]. It should be noted that, basically, the cleavage of H1 by six antibody preparations occurs in clusters containing hydrolysis sites most often after neutral non-charged and nonaromatic AAs: A, V, L, and S (Figure 4, Table 1).

One of the unexpected and completely new results of this study is that antibodies not only against histone H1 but also against H2A, H2B, H3, and H4 are able to hydrolyze histone H1. The main evidence that the preparations of each of the antibodies to all individual histones and MBP do not contain at least noticeable impurities of IgGs against any of the other histones or MBP is that H1 hydrolysis sites for each of these preparations are significantly different (Figure 4, Table 1). In addition, the rate of hydrolysis of H1 histone by IgGs against H2A and H2B is comparable to that for antibodies against H1 histone, but is about 7–10 times lower for the abzymes against H3 and H4. Moreover, in the case of antibodies against H1 (12 sites), H2A (9 sites), and H2B (7 sites), a relatively large number of cleavage sites were found. Abzymes against H3 and H4 hydrolyze H1 histone only at three different sites. It is noteworthy that only a small number of hydrolysis sites by IgGs against different histones completely coincide (Table 1).

As shown previously by the example of hydrolysis of every of five individual histones with antibodies against each of these histones and against MBP from the blood of HIV-infected patients, the main reason for cross-catalysis may be the high level of homology of the sequences of MBP and histones [32,33,34]. Taking this into account, it seemed interesting to analyze the general homology between the protein sequence of H1 with four histones and MBP.

The complete identity of AAs between H1 and H2A (three different alignments) varies from 25.5–26.7% (average value 26.4 ± 1.2%), while similarity (identical together with non-identical amino acids but with highly similar physicochemical properties) varies from 54.7 to 49.7% (average value 52.4 ± 2.5%). Identity between H1 and H2B changes according to three different alignments changes, from 25.3 to 31.5% (average 29.2 ± 3.4%), and similarity from 50.5 to 55.4% (average value 53.5 ± 2.7%). For H1 and H3, the following homology characteristics were found: identity varies between 23.2–28.0% (average value 25.6 ± 3.4%), and similarity between 51.0–51.4% (average value 51.2 ± 0.3%). Approximately the same homology data was obtained for H1 and H4 histones: identity varied between −27.3–31.2% (average 29.2 ± 2.8%), and similarity between −48.6–50.0% (average value 49.3 ± 1.0%). The maximum coincidence of AAs (identity) is observed for H1 with H2B and H4 (29%), while the similarity in H1 sequence with those for the other four histones is comparable (49.3–53.5%). Thus, the homology of the protein sequences H2A, H2B, H3, and H4 with H1 is high and comparable.

An analysis of the homology of the complete sequences of MBP and five histones was carried out: for H1, identity varied between −25.4–28.4% (26.9 ± 2.1%), and similarity between 48.8–52.8% (50.8 ± 2.8%); for H2A, identity varied between −25.0–26.8% (25.9 ± 1.3%), and similarity between 47.6–50.3% (49.0 ± 1.9%); for H2B, only one variant of identity was found: identity −25.9%) and similarity was 52.4%). For H3, identity varied between −22.8–25.3% (24.4 ± 1.4%), and similarity between 44.3–47.6% (45.5 ± 1.8%); for H4, identity varied between −25.0–29.4% (27.2 ± 3.1), and similarity between 46.2–48.6 (47.4 ± 1.1%). The indices of the identity of the AAs of the protein sequence of MBP with those for five histones (24.4–27%), as well as the similarity (45.5–52.4%), are also very close, and almost do not differ from those for H1 and the four other histones. Therefore, these data could explain the possibility of H1 hydrolysis by antibodies against five histones and MBP (Figure 4, Table 1). Due to the high level of homology of the sequences of five histones and MBP, antibodies against these proteins are able not only to form complexes with H1, but also to hydrolyze this histone. In addition, all histones and MBP contain a large number of positively charged residues of lysine and arginine. Such AA residues are necessary for histones’ interactions with negatively charged internucleoside phosphate groups of DNAs. At the same time, it has been shown that MBP is also capable of efficiently forming complexes with DNAs [49]. Thus, it is possible that a large number of positively charged AAs in all histones and MBPs can also make a significant contribution to the ability of antibodies against these proteins to form complexes with foreign histones.

## 5. Conclusions

In this article, we have first shown, on the example of IgGs from patients with multiple sclerosis, that IgGs against H2A, H2B, H3, H4, and myelin basic protein possess an ability similar to anti-H1 IgGs to form complexes with H1 histone, demonstrating polyreactivity in complexation. Moreover, an unexpected result was obtained. IgG-abzymes against H2A, H2B, H3, H4, and MBP possess catalytic cross-reactivity with anti-H1 antibodies, and all of these are capable of hydrolyzing histone H1. Evidence that the ability of IgGs against H1, H2A, H2B, H3, H4, and MBP to hydrolyze H1 histone is their own property follows from the fact that the sites of hydrolysis of H1 histone by different antibodies are individual for each IgG preparation, and differ in their location in the H1 protein molecule. Since histones constantly occur in human blood due to cell apoptosis, the existence of the enzymatic cross-reactivity of abzymes against histones and MBP can play a very negative role in MS pathogenesis.

## Figures and Tables

**Table 1:** Sites of H1 histone hydrolysis by IgGs against hive histones and MBP.

Type of IgGs					
Anti-H1	Anti-H2A	Anti-H2B	Anti-H3	Anti-H4	Anti-MBP
-	**-**	**-**	**-**	**-**	P12-K13 *
-	**-**	**-**	**-**	**-**	***14R-15A***
-	**-**	**-**	**-**	**-**	16K-17A *
-	**-**	**-**	**-**	**-**	**K19-K20**
-	**-**	**-**	**-**	**-**	**K20-S21**
-	**-**	**-**	**-**	**-**	**S21-T22**
**-**	**R46-Q47**	-	**-**	**-**	**-**
**V75-T76**	-	**-**	**-**	**-**	**-**
**-**	**-**	**Q82-T83**	-	**-**	**-**
***R93-L94***	*R93-L94* *	**R93-L94**	-	**-**	**-**
R94-A95		-	-	**-**	**-**
**-**	***L94-A95***	-	-	**-**	**-**
**K96-S97**	-	-	-	**-**	**-**
	-	**S97-D98**	-	**-**	**-**
***A88-S89***	-		-	**-**	**-**
***P100-K101***	**-**	**P100-K101**			**P100-K101**
***S103-V104***	**S103-V104**	-	**-**	**-**	**-**
**-**	V104-A105	-	**-**	**-**	V104-A105
-	**-**	**-**	**F106-K107**	-	F106-K107
-	**-**	**-**	**-**	**-**	***K107-K108***
-	**-**	**-**	**-**	**-**	**K108-T109**
***T109-K110***	**T109-K110**	-	**-**	***T109-K110***	**-**
-	**-**	**-**	**-**	**-**	E112-I113
-					**K115-V116**
-	**-**	**-**	**-**	**-**	**V116-A117**
**A117-T118**	***A117-T118***	-	**-**	**-**	**A117-T118**
-	**-**	**-**	**-**	**-**	**K120-K121**
**-**	***K127-A128***	**K127-A128**	-	**K127-A128**	-
**A128-A129**	-	**-**	**-**	**-**	**-**
K131-A132	-	**-**	**-**	**-**	**K131-A132**
-	***A132-P133***	-	**-**	**-**	**-**
K135-K136	-	**-**	**-**	**-**	**-**
		**K136-P137**		**K136-P137**	
**P141-V142**	-	**-**	**-**	**-**	**-**
-	**-**	**-**	A145-K146	-	**-**
-	**-**	A156-K157	***A156-K157***	-	**-**

* Major hydrolysis sites are marked in bold, moderate in italics, and minor sites in normal. Missing hydrolysis sites are marked with a dash (-).

## Data Availability

The data that supports the findings of this study are available within the article and its supplementary material.

## Supplementary Materials

The following are available online at https://www.mdpi.com/article/10.3390/biom11081140/s1, **Table S1**: Several different medical characteristics of MS patients.

## Abbreviations

AA: amino acid; Ab, antibody; ABZ, abzyme; HIV-1, human immunodeficiency virus type 1; MALDI-TOF, matrix-assisted laser desorption/ionization time-of-flight mass spectrometry; MBP, myelin basic protein; SDS-PAGE, sodium dodecyl sulfate-polyacrylamide gel electrophoresis; MS, multiple sclerosis; SDS-PAGE, sodium dodecyl sulfate-polyacrylamide gel electrophoresis; SLE, systemic lupus erythematosus.
