# Supplementary material for: Multiple Sclerosis: Enzymatic Cross Site-Specific Hydrolysis of H1 Histone by IgGs against H1, H2A, H2B, H3, H4 Histones, and Myelin Basic Protein
Source: Biomolecules. 2021 Aug 2;11(8):1140. doi: 10.3390/biom11081140 (PMC8392522; doi:10.3390/biom11081140)
Supplement: Supplementary file 1 [file biomolecules-11-01140-s001.zip › biomolecules-1310478-supplementary.pdf]

Supplementary Table S1.

In this work, from IgG samples of 59 patients with multiple sclerosis, which were described earlier in [31], 15 preparations having increased activity in the hydrolysis of histone H1 and MBP were selected to obtain a mixture of IgG preparations. The medical indexes of 57 are given in [31] and of 15 patients are shown below. The mixture of IgG preparations was used for preparation IgGs against H1, H2A, H2B, H3, H4, and myelin basic protein described in this work.

Several different characteristics of MS patients

№	MS subtypes	Sex	Age (years)	Disease duration (years)	EDSS*
Debut of multiple sclerosis (DMS)**					
1	DMS1	F	26	0.083	3.0
2	DMS2	F	24	0.17	4.5
3	DMS3	F	21	0	2.0
4	DMS4	M	30	0	3.5
5	DMS5	M	29	0	3.5
6	DMS6	F	43	0	4.0
Remitting multiple sclerosis (RMS)					
7	RMS1	M	46	16	4.0
8	RMS2	F	22	1	3.5
9	RMS3	F	53	23	3.5
10	RMS4	F	38	12	3.0
11	RMS5	F	58	20	3.0
12	RMS6	M	38	9	4.0
13	RMS7	F	45	11	4.0
14	RMS8	F	34	1	3.5
15	RMS9	F	55	10	3.5
Average values	15 patients	11 F and 4 M	37.5 ±12.2	6.9 ±8.1	3.5 ±0.6

*Kurtzke's expanded disability status scale (EDSS)

**Debut of multiple sclerosis (DMS) corresponds to the first coming of patients in the clinic for research after the early manifestations of signs of this pathology
